# Our pride, our joy: An intersectional constructivist grounded theory analysis of resources that promote resilience in SGM communities

**DOI:** 10.1371/journal.pone.0280787

**Published:** 2023-02-03

**Authors:** O. Winslow Edwards, Eliot Lev, Juno Obedin-Maliver, Mitchell R. Lunn, Micah E. Lubensky, Matthew R. Capriotti, J. J. Garrett-Walker, Annesa Flentje

**Affiliations:** 1 Faculty of Health Sciences, Simon Fraser University, Vancouver, British Columbia, Canada; 2 Department of Community Health Systems, School of Nursing, University of California, San Francisco, California, United States of America; 3 The PRIDE Study/PRIDEnet, Stanford University School of Medicine, Stanford, California, United States of America; 4 Department of Epidemiology, Rollins School of Public Health, Emory University, Atlanta, Georgia, United States of America; 5 Department of Obstetrics and Gynecology, Stanford University School of Medicine, Stanford, California, United States of America; 6 Department of Epidemiology and Population Health, Stanford University School of Medicine, Stanford, California, United States of America; 7 Department of Medicine, Division of Nephrology, Stanford University School of Medicine, Stanford, California, United States of America; 8 Psychology Department, San Jose State University, San Jose, California, United States of America; 9 Department of Applied Psychology and Human Development, Ontario Institute for Studies in Education, University of Toronto, Toronto, Ontario, Canada; 10 Department of Psychiatry, Alliance Health Project, School of Medicine, University of California, San Francisco, California, United States of America; Charles Sturt University - Port Macquarie Campus, AUSTRALIA

## Abstract

**Introduction:**

Sexual and gender minority (SGM) communities, including lesbian, gay, bisexual, transgender, queer, intersex, asexual, and Two-Spirit people, have historically been researched from a deficits-based approach that fails to highlight the ways communities survive and thrive in the face of adversity. This study endeavored to create a model of resources that promote SGM resilience using a sample that amplified traditionally underrepresented perspectives, including individuals from racial and/or ethnic minority groups, trans and/or gender diverse individuals, individuals on the asexual spectrum, and older adults.

**Methods:**

Participant responses to three open-ended questions from The PRIDE Study’s (an online national longitudinal cohort study of SGM people) 2018 Annual Questionnaire were analyzed using constructivist grounded theory. These questions examined what brings people joy and what they appreciate most about their SGM identity. Participants (n = 315) were randomly selected from a larger sample of people who had responded to demographic questions and at least one open-ended question (N = 4,030) in a manner to ensure diverse representation across race/ethnicity, gender identity, sexual orientation, age, and region of residence.

**Results:**

The proposed model includes social resources (Connecting with Others, Cultivating Family, Helping Others, Participating in Culture and Spirituality), affective generative resources (Engaging in Enriching Pursuits, Accessing Economic Resources), and introspective resources (Exploring One’s Authentic Self, Persevering through Hardship) that are theorized to contribute to SGM resilience across the life course.

**Conclusions:**

SGM communities may tap into various resources to promote resilience. As public health practitioners, we can help to foster this resilience by resourcing and supporting initiatives that foster social connection, create spaces for community members to engage with various types of enrichment, facilitate access to economic resources, and provide support and inclusion for all SGM community members.

## Introduction

Sexual and gender minority (SGM) communities–such as lesbian, gay, bisexual, transgender and/or gender diverse, queer, intersex, asexual, and Two-Spirit people–have historically been researched from a deficits-based approach that centers problems, risky behaviors, and pathologies [1–3]. Although this research has made important contributions to the field, it may add to stigma by emphasizing deficiencies among SGM individuals and using cisgender, heterosexual people as ‘the standard’ to which SGM health is compared [4]. As such, researchers have increasingly advocated for the use of strengths-based approaches, which focus on the resources that SGM communities utilize to persist and thrive despite stigma and adversity [1–4].

### Sensitizing frameworks

According to the Minority Stress Model, SGM individuals experience unique stressors as a result of societal stigma, which can lead to adverse health outcomes [5,6]. Meyer’s Minority Stress Model accounts for response to stress through coping, which refers to any effort one makes to positively adapt to stressors that may attenuate the effects of minority stress [6]. Resilience describes successful coping and ‘bouncing back’ after experiences of adversity [4,7,8]. In alignment with the recent emphasis on strengths-based approaches, resilience has become an integral concept in shifting the research agenda to highlight the myriad resources that SGM communities draw upon to foster their health and wellbeing [2,7].

Resilience as a framework has been criticized for its individualistic focus arising from a Western and Eurocentric perspective [1]. Viewing SGM communities through an intersectional lens acknowledges that SGM individuals have heterogenous lived experiences of structural privilege and oppression that vary with social identifiers including race, ethnicity, gender identity, sexual orientation, age, disability, and class, among others [1,2,4,9,10]. Per Crenshaw’s seminal Black feminist critique of the framing of race and gender as mutually exclusive, intersectionality describes the way structural factors–such as racism, settler colonialism, hetero- and cis-normativity, sexism, ageism, ableism, and classism–interact and overlap to shape individuals’ social environments [7,9,10]. When considering resilience within SGM communities, an intersectional lens considers the various impacts of these interdependent structural factors on experiences of coping and resilience [10]. Research informed by an intersectional lens often draws from a diverse sample to illuminate varied and/or underrepresented lived experiences of SGM community members, such as individuals who are from a racial or ethnic minority group, individuals who are transgender and/or gender expansive and individuals on the asexual spectrum [1,2,4,10]. Notably, within the framework of intersectionality, social identities are understood to be interdependent instead of additive, i.e. the sum of one’s experience cannot be understood by separately considering their race in addition to their ethnicity, their gender identity, their sexual orientation, etc.; their experience should be understood through the consideration of their various social identifiers collectively [2,10].

Thus, researchers have suggested that resilience as a framework should integrate an intersectional lens, conceive of coping and resilience as ongoing processes, and consider resilience-promoting factors on a continuum between individual- and community-level resources [1,2,7]. To clarify the scope of community level resilience, Meyer [2015] emphasizes that SGM individuals exist within social environments and communities that can bolster individuals by providing access to both tangible and intangible resources that promote resilience, including the “[reframing of social] norms and values, role models, and opportunities for social support” [p. 211].

Fredrickson’s broaden-and-build theory asserts that certain positive emotions–such as joy, pride, and love–can broaden an individual’s planning repertoire and build up enduring, beneficial psychological and social resources that can be employed to address future adversity [11]. Indeed, research demonstrates that experiencing joy or positive emotions can decrease reactivity to stressors and support the ability to rebound from stress [12,13]. Within SGM communities, positive SGM identity–specifically feeling pride in, appreciating and accepting attributes associated with one’s SGM identity–has also been linked to higher reported life satisfaction and lower severity of reported depressive symptoms [14–16]. Thus, investigating contributors to joy and positive SGM identity can serve as a basis for conceptualizing resources that may promote resilience within SGM communities.

Recent qualitative research has examined resilience and/or coping resources in the context of SGM youth [17–19], SGM adults [20–23], and SGM older adults [24,25]. This research, however, has primarily focused on specific SGM subgroups (*i*.*e*., transgender school psychologists, gay Latino men, bisexual older adults), employed small sample sizes, and been limited in racial/ethnic diversity. An important next step is to examine resources that may promote resilience within a larger, age-varied, and racial/ethnically diverse sample that is inclusive of many different SGM communities.

The purpose of this study was to employ an intersectionally-informed strengths-based, constructivist grounded theory approach to explore joy and positive SGM identity, both conceptualized as major contributors to coping and resilience among SGM people. As this study did not evaluate any health outcomes, we investigated probable resources that can promote resilience as described by SGM people. We did this by examining open-ended responses to three questions: what brings individuals joy, how they positively relate to their sexual orientation, and how they positively relate to their gender identity. The questions about sexual orientation and gender identity asked participants to reflect on a single aspect of their sexual and/or gender identity. Despite this framing, we chose to analyze the data through an intersectional lens that considered the impacts of structural factors on participant experiences. To facilitate this, we amplified underrepresented voices via stratified sampling strategies to support a more thorough examination of coping and resilience within a diverse sample.

## Methods

### Participants

Participants were from The Population Research in Identity and Disparities for Equality (PRIDE) Study, a longitudinal cohort study of the general health of SGM adults in the United States (US) [26]. Inclusion criteria for The PRIDE Study were: (i) being at least 18 years old at the time of enrollment, (ii) identifying as a sexual and/or a gender minority person, (iii) residing in the US or its territories, and (iv) comfort with reading and writing in English [26].

Participants who answered at least one of the relevant open-ended questions and completed demographic questions (N = 4,030) were sampled for this study. Using STATA (Version 13), the sample was initially stratified by sexual orientation, gender identity, and race/ethnicity to create equivalent representation of diverse participant groups and ensure inclusion of historically underrepresented voices in SGM research (*i*.*e*., racial and/or ethnic minority participants, bisexual and pansexual participants, asexual spectrum participants, and transgender and/or gender expansive participants). Regarding sexual orientation, Manago [27] coined same-gender loving as a culturally-affirming descriptor for same-gender and multi-gender attracted people of the African diaspora. Because participants self-selected their sexual orientation(s), individuals outside of the African diaspora also identified as same-gender loving which initially resulted in a sample of same-gender loving participants that was primarily composed of non-Black respondents. See **S1 Appendix** for information about how our sampling strategy addressed this to ensure representation of same-gender loving individuals of the African diaspora.

Per the initial stratification, participants who had not responded to sexual orientation, gender identity, and race/ethnicity were not sampled. From this group, 80 participants total were randomly sampled from the aforementioned 3 strata (*i*.*e*., sexual orientation, gender identity, and race/ethnicity) using STATA’s ‘sample’ function. Iterative purposive sampling occurred to enrich the sample for participants with underrepresented ages, regions and sexual orientations, while maintaining stratification by race/ethnicity (S1 Appendix). As rich or detailed description is important for robust qualitative analyses [28], responses of longer than average character length were also sampled during iterative purposive sampling to ensure the exploration of sufficient data to support analytic conclusions. Two participants with uncodeable text responses to one of the open-ended questions were excluded from analysis (n = 315) (Table 1). Theoretical saturation was determined when new codes were no longer noted, and the coders determined that categories had been well defined and explored within the scope of the data [28].

**Table 1:** Sample characteristics.

Race/Ethnicity^1^	n = 315	% of Total
American Indian or Alaskan Native	36	(11.4%)
Asian	35	(11.1%)
Black, African American or African	37	(11.7%)
Hispanic, Latin American or Spanish	59	(18.7%)
Middle Eastern or North African	12	(3.8%)
Native Hawaiian or other Pacific Islander	3	(1.0%)
White	250	(79.4%)
More than one	101	(32.1%)
**Gender Identity Grouping**		
Cisgender man	86	(27.3%)
Cisgender woman	77	(24.4%)
Gender-expansive	77	(24.4%)
Transfeminine	39	(12.4%)
Transmasculine	36	(11.4%)
**Intersex** ^**2**^ ^,^ ^**3**^		
Yes	6	(1.9%)
**Sexual Orientation** ^**1**^		
Asexual spectrum	52	(16.5%)
Bisexual	83	(26.3%)
Gay	113	(35.9%)
Lesbian	65	(20.6%)
Pansexual	67	(21.3%)
Queer	139	(44.1%)
Questioning	16	(5.1%)
Same-gender loving	49	(15.6%)
Straight/Heterosexual	9	(2.9%)
**Age**		
18–24	54	(17.1%)
25–34	96	(30.5%)
35–49	51	(16.2%)
50–64	64	(20.3%)
65+	50	(15.9%)
**Region** ^**2**^		
Northeast	56	(17.7%)
Midwest	60	(19.0%)
South	76	(24.1%)
West	120	(38.1%)
Missing	3	(1.0%)
**Income** ^**2**^		
$0	19	(6.0%)
$1 - $30,000	125	(39.7%)
$30,000 - $60,000	76	(24.1%)
$60,000 - $90,000	39	(12.4%)
$90,000+	46	(14.6%)
Missing	10	(3.2%)
**Education** ^**2**^		
Nursery school to high school, no diploma	2	(0.6%)
High school graduate or equivalent (*e*.*g*., GED^4^)	10	(3.2%)
Trade/Technical/Vocational training	6	(1.9%)
Some college	71	(22.5%)
2-year college degree	13	(4.1%)
4-year college degree	93	(29.5%)
Master’s degree	7	(2.2%)
Doctoral degree	23	(7.3%)
Professional degree (*e*.*g*., M.D., J.D., M.B.A.^4^)	20	(6.3%)
Missing	70	(22.2%)

^**1**^ Categories were not mutually exclusive, as such percentages do not add to 100%.

^**2**^ Not all participants responded, declining to answer was allowed.

^**3**^ Based on response to a yes/no question asking whether the participant identifies as intersex.

^4^ GED–General Education Development (high school equivalency test), M.D.–Medical Doctor, J.D.–Juris Doctor, M.B.A.–Master of Business Administration.

The PRIDE Study was approved by the Institutional Review Boards of the University of California, San Francisco and Stanford University. Electronic informed consent was obtained prior to participation in The PRIDE Study through the online participant portal. Theoretical coding and conceptual model development was approved by Simon Fraser University’s Research Ethics Board.

### Questions

We analyzed responses to three questions from the 2018 Annual Questionnaire of The PRIDE Study: (1) “We at The PRIDE Study are interested in what makes people thrive. Therefore, can you tell us a bit about what brings you joy?”, (2) “We are excited to know about people’s positive experiences in relation to their sexual orientation! Please tell us what you most like about being or are most proud of being gay/lesbian/bisexual/or a sexual minority,” and (3) “We are excited to know about people’s positive experiences in relation to their gender identity! Please tell us what you are most proud about being genderqueer/transgender/gender non-binary/or a gender minority.” Questions 2 and 3 were shown based on participant preference to see questions designed for sexual minority people, gender minority people or both.

### Analysis

We employed a constructivist grounded theory approach to analyze the data. Grounded theory is a qualitative methodology that facilitates the development of theories grounded in extant data, while constructivism considers researcher ‘objectivity’ and acknowledges the shared role of investigators and participants in co-constructing meaning from the data [28]. Grounded theory is flexible to the researchers’ unique needs in navigating data analysis [29] and was used here to analyze open-ended responses to survey questions, as in Bogetz et al. [30], Gaffney et al. [31], and Riggle et al. [32].

In alignment with constructivist grounded theory, it is important to situate those with direct involvement in data analysis. The first author, O. Winslow Edwards (OWE), served as the primary researcher for this project and analyzed participant responses in close collaboration with Eliot Lev (EL). OWE is a neurodivergent, nonbinary queer person with primarily Afro-Jamaican, Chinese Jamaican, Indo-Jamaican, and White European settler ancestry. They bring an intersectional, anti-oppressive public health lens that focuses on the social determinants of health. EL is a Jewish immigrant from Russia and a transgender and sexual minority person with disabilities. He brings a multicultural humanistic feminist framework with focus on mental health. OWE and EL take the role of insider-outsiders in this study; while they both situate themselves as community members who share experience with the topics studied, they also have unique identities that do not overlap with the panoply of lived experiences of diverse SGM community members [33].

The PRIDE Study Research Advisory Committee (RAC) designed the questions analyzed in this study using community-based participative approaches. The Participant Advisory Committee (PAC) provided feedback, critiques, and insight integral to model development and sampling approaches. Both the RAC and PAC are comprised of a diverse group of SGM individuals.

Dedoose (Version 8.3.35) online qualitative analysis software was used for the coding process. Initial coding started by considering line-by-line data units, examining the content of sentences and utilizing gerunds to create active codes (i.e. being in affirming community, wanting to live authentically) [28]. Constant comparative methods were used throughout to define and probe the meanings of codes through comparison with other codes as novel data was analyzed [28]. Coders (OWE and EL) participated in memo writing over the course of the project to contemplate conceptual issues and explore the content, differences, and similarities of codes [28]. OWE and EL coded the same first ten responses separately and then came together to compare code application and create an initial codebook. After this, OWE and EL coded separately such that each coded half of the remaining responses. Following coding of 80 participants’ responses, coders convened to review and agree upon code definitions for a comprehensive codebook.

Finally, focused coding ensued wherein salient codes were applied to large amounts of data, with coders meeting regularly to discuss coding practices, review memos, make changes or updates to the codebook, and discuss emergent questions. Following focused coding, OWE undertook theoretical coding through mind-mapping, memo writing, and creating visual representations of a conceptual model to explore the relationships between focused codes [28].

## Results

Demographic characteristics of participants can be found in Table 1. Race/ethnicity was not mutually exclusive and 32% of participants indicated more than one race/ethnicity category. By magnitude, 79% of participants reported White race/ethnicity, 19% Hispanic, Latino or Spanish, 12% Black, African American or African, 11% American Indian or Alaskan Native, 11% Asian, 4% Middle Eastern or North African, and 1% Native Hawaiian or other Pacific Islander.

Gender identity was mutually exclusive: cisgender participants reported that their gender was the same as their sex assigned at birth, transfeminine and transmasculine participants reported that their gender was different than their sex assigned at birth and aligned with a feminine binary identity (*e*.*g*. woman, girl, transgender woman) or a masculine binary identity (*e*.*g*. man, boy, transgender man), and gender expansive participants reported a non-binary gender identity (*e*.*g*. agender, genderflux, nonbinary) or multiple gender identities that were not reflective of the same binary gender irrespective of sex assigned at birth. Per the aforementioned groupings, 27.3% of participants were cisgender men, 24% were cisgender women, 24% were gender-expansive people, 12% were transfeminine people, and 11% were transmasculine people. Among all participants, 2% identified as intersex.

Sexual orientation was not mutually exclusive and 44% of participants identified as queer, 36% as gay, 26% as bisexual, 21% as pansexual, 21% as lesbian, 17% as asexual spectrum (e.*g*. ace, grey, demisexual), 16% as same-gender loving, 5% as questioning, and 3% as straight. Sampling occurred across all adult ages and 52% of participants were aged 35 or older. Sampling occurred across all regions in the US, with the largest participant representation in the West (38%) and the South (24%). Considering income, 27% of participants reported an annual income of $60,000 or higher and 49% of participants held a postsecondary degree, such as a college degree, graduate degree, or professional degree.

**Fig 1:**
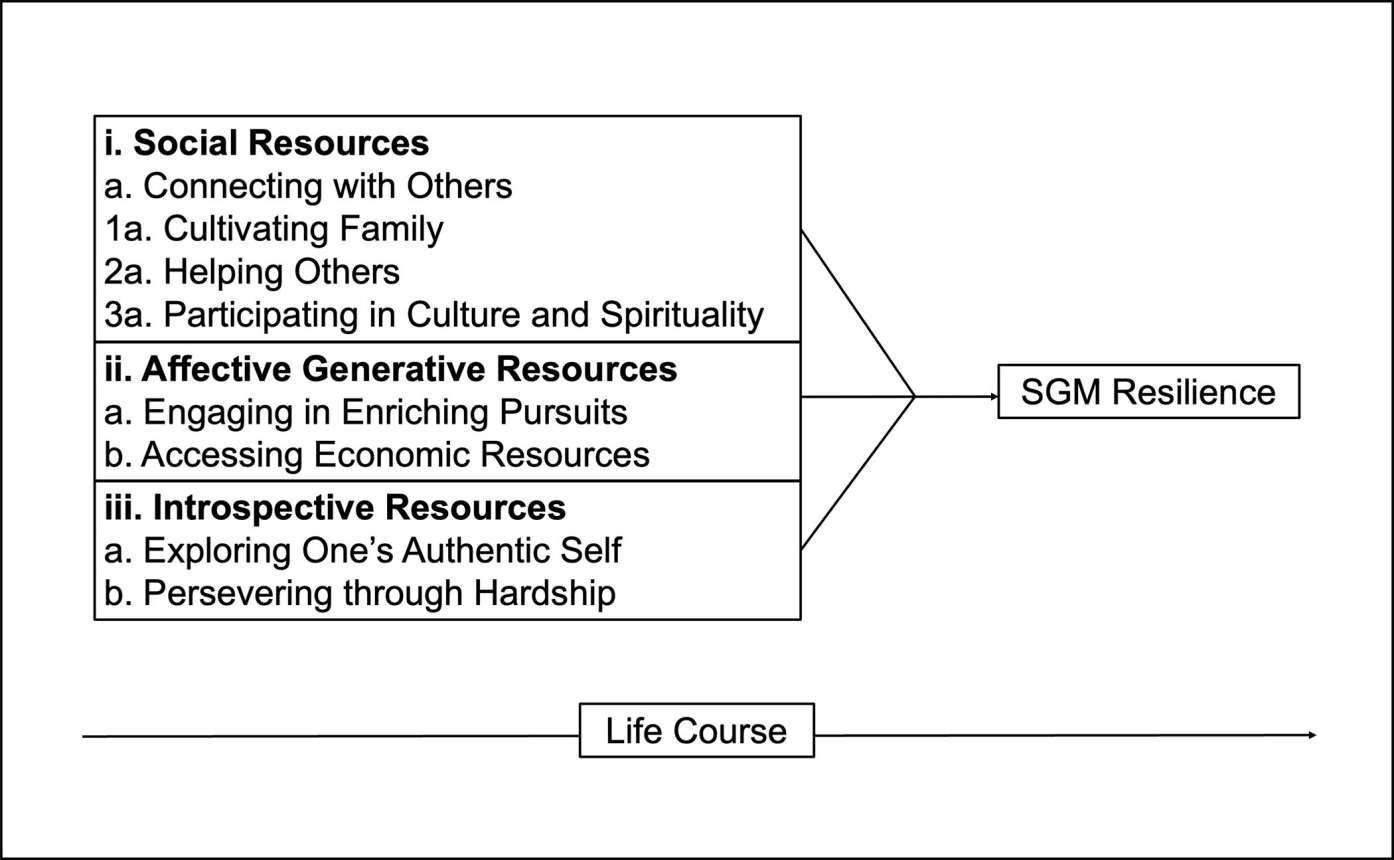
Proposed model of resources that promote SGM resilience.

The proposed model of resources that support SGM resilience can be broken down into three groupings (social, affective generative, introspective), listed in arbitrary order, which encompass eight resources (Fig 1). This model was generated and emerged from theoretical coding. The social resources include different types of social and relational connection that were important to participants; the affective generative resources identify activities and resources that contribute to joy and wellbeing; while the introspective resources identify internal processes of self-exploration, endurance through negative experiences, and growth navigated by participants related to SGM identity and other salient social identities. These resources are theorized to overlap and interact with one another across the life course. Certain resources included similar responses across race and ethnicities, gender identities, sexual orientations, and ages, while others included aspects that were specific to particular sub-populations.

Within the following quotations, responses have been unmodified except when adding [*sic*] to identify typos and brackets to clarify meaning or reduce identifiable information within the quote. Bracketed text has been underlined to indicate when identifiable information has been withheld.

### Social resources

#### Connecting with others

*Connecting with Others* took place when individuals interacted with and/or formed relationships with others. When connection occurred, it was described as a process that brought feelings of joy and support as well as helping to link respondents to their authentic SGM identity. On the other hand, lack of connection or negative experiences of social and/or familial rejection were tied to feelings of loneliness, frustration, and desire for social connection (Table 2) (see below, *Persevering through Hardship*).

**Table 2:** Model resources and example responses.

Category	Domain/Sub-Domain	Exemplar Responses^1^
Social Resources	Connecting with Others	Given where I live there isn’t a very strong LGBT+ community, so I’ve had to turn to online communities (like Tumblr) and there are very supportive communities that I’ve found. *19*, *Hawaiian Native/Pacific Islander*, *cisgender woman*, *pansexual* I also crave good human company, but I don’t get much of that, living alone and in the neighborhood where I bought my home. *63*, *White*, *cisgender man*, *gay*
	Cultivating Family	The ‘extended family’ one choices [*sic*] to adopt and be adopted by gives one a sense of community and loyalty. *66*, *Hispanic*, *cisgender woman*, *lesbian* Spending time with my friends and family brings me joy. My cat brings me joy. *47*, *White*, *cisgender man*, *gay* I love my girlfriend and how queer we are as a couple. I love love; I’m a romantic. *21*, *White*, *gender expansive person*, *asexual spectrum*, *pansexual*, *queer*
	Helping Others	Helping my community through my peer support group. Volunteering at a food pantry. *34*, *White*, *gender expansive person*, *gay*, *pansexual*, *queer* Also I’ve had good lesbian role models my whole life because my aunts are married and run a [farm name] together and I’ve helped there every summer since I was 8. Watching how settled and comfortable and in love they are is always wonderful and makes me proud that I might get a love like that someday. *19*, *Asian*, *White*, *gender expansive person*, *lesbian*, *queer* Seeing older out lesbians of color[.] Having mentors that look like me and are also out[.] *34*, *Asian*, *cisgender woman*, *gay*, *lesbian*, *queer*
	Participating in Culture and Spirituality	Being indigenous, there is a concept within my racial community of being ‘twospirit’ [*sic*]—that gender non-conforming folks aren’t some kind of a mistake, but that we were put here to bridge the gap between men and women. Historically, for many tribes, twospirit [*sic*] people were viewed as sacred, and looked to for advice. Having both ‘male’ and ‘female’ spirits—two spirits—meant that we were/are seen as being able to view things from either perspective. *19*, *American Indian/Alaskan Native*, *White*, *gender expansive person*, *bisexual*, *pansexual*, *queer*, *same-gender loving* I have experienced wonderful community life as a member of a gay and lesbian synagogue. *68*, *White*, *cisgender woman*, *lesbian* I always focus on gratitude for all the good things I’ve been able to do, or feel, or learn. I have a practice of daily gratitude instead of meditation or prayer. I find something positive in every situation. I look for the goodness in everyone—it’s there. I do not dwell on the past hurts or setbacks, or pain. I am either in the present or in the future, thinking positive thoughts. *68*, *White*, *Hispanic*, *cisgender woman*, *bisexual*, *lesbian*, *queer*
Affective Generative Resources	Engaging in Enriching Pursuits	I love watching sports, especially soccer (Portland Thorns and Timbers), baseball (Pittsburgh Pirates), hockey (Pittsburgh Penguins), and basketball (Portland Trailblazers). I love drawing and writing fiction, poetry, and music. I enjoy playing flute in musical ensembles. I love playing D&D with a bunch of my queer friends, and I love spending time with them too. *19*, *Asian*, *White*, *gender expansive person*, *lesbian*, *queer* New experiences are my reason for living. I write and I read widely. I ride a motorcycle and am learning to play the guitar, because if I don’t fulfill classic lesbian stereotypes, who will? I love music of all sorts, I travel widely, I meet as many people of different experiences as I can. I want to do everything, see everything. It’s the driving force that gets me up in the morning and allows me to power through depression. *24*, *White*, *transfeminine person*, *gay*, *lesbian*, *queer*
	Accessing Economic Resources	Thinking about being able to afford FFS [facial feminization surgery]. *31*, *American Indian/Alaskan Native*, *White*, *transfeminine person*, *pansexual*, *bisexual*
Introspective Resources	Exploring One’s Authentic Self	Being Genderqueer has not only given me the awareness to scrutinize the gender norms of society, but also scrutinize the values I have toward gender and sexuality. This introspection of my own values and identity has allowed me to look at the world with a unique view. I am hyper-aware of disparities and inequalities faced by sexual, gender, and racial minorities. Because of my gender identity, I not only have a responsibility to help remedy these disparities, but also a passion and drive to contribute to the work that seeks to end these forms of discrimination. It would be untrue to say that I have not suffered for my gender identity; but it would be a grave oversimplification to say that my life would be better had I been gender conforming. Yes, my life may be more difficult because of my gender identity. But my life is also far richer because of my gender identity, not despite it. To paraphrase Nietzsche, “It is the growth from pain and hardship that gives meaning to life.” *25*, *Asian*, *gender expansive person*, *gay* I walk everyday in my truth. Being authentic has helped me to be a much happier better me. *50*, *Black*, *transmasculine person*, *gay*, *same-gender loving* I’ve put a lot of work into loving myself, through therapy, and self-care practices. My sexuality is a very important part of who I am. It makes me unique, and I’d rather be unique than the same as everyone else. Valuing that uniqueness and diversity has enriched my life in so many ways. *47*, *White*, *cisgender man*, *gay*
	Persevering through Hardship	At first, I was both terrified and disgusted with myself for failing to be the “man” my deeply religious family expected me to be. I am very proud to have come to my senses and realized that I was born the way I am and that I don’t have to accept the shame my family demanded that I endure at their hands… I am proud of the fact that I am nolonger [*sic*] ashamed of who I am. I’ve accepted ME [*sic*] and I’m in transition to be more myself than how I was born. *63*, *Multiracial*, *Hispanic*, *transfeminine person*, *queer*, *lesbian*, *gay* It took me years to accept my sexual orientation because of my religious upbringing, but now I am so proud of it. Accepting my sexual orientation allowed me to stop hating myself for loving my best friend. Accepting my sexual orientation allowed me to have a wonderful, deep, and intimate relationship with my late partner, who was the most amazing person I’ve ever met. My only regret is that it took me so long to fully accept myself. *29*, *White*, *cisgender woman*, *queer* Being trans and a person of color is difficult, but it’s also a great feeling to feel congruent… also white [SGM] people can be terribly racist. *33*, *Asian*, *transfeminine person*, *lesbian*

^1^ Quotes have been unmodified except adding [*sic*] to identify typos and when brackets were added for meaning or to reduce identifiable information within the quote. Bracketed text has been underlined to indicate when identifiable information has been withheld.

Respondents exemplified how connecting with others often provided a sense of warmth, community belonging, and acceptance, whether that connection was in-person or online (Table 2): “I see how beloved I am in this community directly or indirectly and all of the loneliness and coldness I feel towards people like me melts away” (22, Black, cisgender woman, queer, questioning, same-gender loving). Social relationships with other SGM people were important, and many respondents felt affirmed by their membership in the SGM community at large: “I’ve also had the opportunity to meet some very great people at gay bars, gay sports leagues, and other gay social activities, and as such, I feel like I belong to something bigger; a community of awesome people” (31, White, Hispanic, cisgender man, gay).

Multiple participants who were Black or a person of color described the importance of sharing community with other SGM people of color: “I’m proud of having more and more Black faces coming forward as Bi+ and seeing them come into their own and they grown [*sic*] in their identities. I’m proud to be in an ever growing, ever evolving community” (27, Black, cisgender woman, bisexual). This was especially important considering the racism experienced by many participants (see *Persevering through Hardship*).

*Connecting with Others* is a higher-level resource that is inclusive of the following three resources, which highlight particular connection types that were especially salient to participants.

1a. Cultivating Family

*Cultivating Family* acknowledged the focal role of family of origin and/or chosen family in providing love, fulfillment, and companionship in respondents’ lives: “I have been very fortunate to have a [*sic*] great support from family and friends all my life” (57, Hispanic, cisgender man, gay). As in the above quote and the exemplars (Table 2), many felt accepted by and enjoyed spending time with their family of origin. For others, chosen family provided the encouragement, validation and understanding that may have been lacking from relationships with one’s family of origin: “I know that no matter what happens with my relationships with my work colleagues and my family, I know that I have my queer community to fall back on, and that they will always accept and support me” (25, Asian, White, gender expansive person, gay, queer, lesbian). Though this participant may experience rejection or inconsistency in their relationships with coworkers and family members, they can depend on their queer community for encouragement and affirmation. *Cultivating Family* was often linked to one’s ability to authentically express their SGM identity, and many respondents expressed appreciation that their friends and/or family acknowledged and accepted them for who they are: “I love being a part of such a loving and diverse community. I’m accepted and free to be myself with my LGBT+ friends” (27, White, cisgender woman, asexual spectrum, bisexual, pansexual, queer).

For some, family was inclusive of pets and animals, who provide love and companionship (Table 2). As one participant explained, “It’s… a rough time in my life for me. (We’re talking ‘second home-disrupting disaster in six months combined with severe career burnout’ rough.) So here are a few of the bright spots: my spouse who has my back, 10000%… my kitten who washes my face and purrs… my dog who loves me and leans on me and wants me around” (28, White, gender-expansive person, asexual, queer). Feeling care, love, and a desire to spend time together from their spouse and companion animals comforted this participant as they navigated an exceptionally stressful time in their life.

Respondents of all ages emphasized the importance of friends, family, and animals in their lives, though the types of relationships highlighted varied some by age. Respondents aged 35 and up more often highlighted relationships with spouses, grandchildren, children, and nieces or nephews, while younger respondents more often discussed dating relationships (Table 2). Exemplar responses demonstrated the importance of various types of family and the satisfaction that positive relationships provide (Table 2): “Being loved and feeling loved. Having a long-term healthy relationship, 19 years, being a daddy to two wonderful little girls… Feeling lucky that I get to be a husband, lover, and the primary caretaker/mommy to my children. I made my dreams come true” (54, Multiracial, Hispanic, gender expansive person, gay). For this participant, maintaining a loving, long-term partnership and nurturing their children as a primary caretaker was the extremely rewarding culmination of a major aspiration. Friendship and animal companionship were very important to participants of all ages.

1b. Helping Others

*Helping Others* included altruistic activities such as volunteerism, activism, and working in caregiving professions or other positions that allow one to make a difference. Activism presented in many ways, such as participating in political activism, engaging in community outreach, and teaching the broader community about SGM identities (Table 2). One participant described the fulfilment they get from volunteering: *“*We just had our city’s Pride Festival, and one of the things that really brings me joy is being behind the Bi [Southeastern state] table and talking with folks as they find out they are not alone! I love doing outreach and education…” (61, White, gender expansive person, bisexual, queer). *Helping Others* made respondents feel that they were contributing or giving back for the benefit of the SGM community or the community at large.

Many discussed their experiences with mentorship or role modeling, including ways they had individually supported the development of other community members and/or the ways others had contributed to their ability to understand themselves and live authentically (Table 2): “I get to tell younger people that their feelings are normal, their emotions and attractions are normal, because I definitely did not get that reassurance when I was a child” (28, American Indian/Alaskan Native, gender expansive person, bisexual, pansexual, queer). Providing reassurance and affirmation allowed this participant to counteract the societal messaging of hetero- and cis-normativity for SGM youth who are discovering themselves. Another participant of color highlighted the importance of having role models in the community who look like her (Table 2).

1c. Participating in Culture and Spirituality

This resource included engagement with cultural activities, beliefs, and spiritual practices identified by respondents. Cultural beliefs were highlighted that positively connected respondents’ SGM identity to their heritage. For multiple participants, connecting to their cultural and/or ethnic heritage provided an affirming perspective toward their gender identity, as in the following exemplar: “My genderqueerness is one aspect of my self [*sic*] that makes me (a Jewish person) feel a connection to my ancestors. Torah recognizes other genders, so not only am I fine in the eyes of HaShem, not only am I made B’tzelem Elohim, but I am also included in the long tradition of my people. I may not use the specific words, but I feel their spirit in me” (19, Asian, White, gender expansive person, lesbian, queer). Many participants shared similar stories demonstrating links between cultural concepts and SGM identities that brought a sense of belonging and connection (Table 2)

Other participants discussed participating in spiritual, religious, and/or cultural communities and the feeling of happiness and kinship this can provide (Table 2): “It took time to get here, but today I’ve found community, connection, authenticity, integrity and hope. I am in a queer relationship with someone I love beyond words and a leader in a religion that is working hard to support LGBTQ+ people” (43, American Indian/Alaskan Native, White, gender expansive person, queer, pansexual). Finally, many described practices of yoga, meditation, and gratitude as practices that foster positivity and joy (Table 2).

### Affective generative resources

#### Engaging in enriching pursuits

*Engaging in Enriching Pursuits* described varied activities that inspired happiness and positive emotions for respondents. Common pursuits included engaging with nature, exercising, participation in creative activities (*e*.*g*., writing, theatre, cooking), interacting with media (*e*.*g*., television, video games, books), and being mentally stimulated (*e*.*g*., learning new things) (Table 2), as shown in the following response: “Since 2013 I’ve kept up a moleskin journal. Even when I dont [*sic*] have answers for my struggles it’s a comfort and a pleasure to pick up a pen and write it all away. For nearly three years now I’ve been taking boxing classes and for nearly two years I’ve been taking violin lessons. I have enjoyed the challenges and achievements that both have brought me” (27, Black, cisgender woman, bisexual). Many enriching pursuits were done individually (*e*.*g*., reading, writing, self-care), while others occur in community (*e*.*g*., playing board games, singing in a chorus, having friends over for dinner).

For some participants, their job was a source of pleasure and satisfaction. As one participant described, “I am fortunate to have a job at which I excel and which brings me tremendous joy and pride. I manage and teach at a mixed martial arts school, and I have seen my programs change people’s lives. This is fun, strenuous, exhausting, exhilarating work and I am grateful every day that I get to do it” (38, White, cisgender woman, bisexual, gay, lesbian, queer). For this participant, her job allows her to be physically active and to engage in the creative process of teaching, while also tapping into *Connecting with Others* more broadly and *Helping Others* by facilitating change in the lives of her students. Taken together, this respondent’s job provides a continual source of happiness, gratitude in her life. Considering that many full-time positions require at least 40 hours weekly to be spent working, being able to tap into pursuits such as being physically active, being creative and feeling mentally stimulated during one’s job may greatly contribute to positive emotions.

As suggested in the following response, engagement in these activities may bolster one’s positive ‘emotional reserves’ to deal with difficult experiences: “Learning about anything and everything, but especially science and history. And the little daily pleasures like video games, movies and documentaries, tasty food, seeing a bit of nature in the city, clean bedsheets, masturbation, music, laughing at dumb jokes… stuff that adds up to make me think ‘I live a happy, pleasurable everyday life.’ I find that so important for forming a foundation of calmness and emotional reserves in order to tackle the difficult stuff” (30, Asian, White, transmasculine person, asexual spectrum, gay, queer). Similar to another exemplar (Table 2), these respondents suggest that the ability to engage with *Enriching Pursuits* and the presence of joy supports coping and the ability to bounce back or persevere through ‘the difficult stuff.’

#### Accessing economic resources

Various participants discussed or alluded to the importance of *Accessing Economic Resources*, specifically having access to safe and reliable employment or other financial support. Within the US, employment provides access to money and often health insurance, thus acting as an important facilitating factor that allows individuals to engage with many enriching pursuits, access health and gender-affirming care, afford medical copayments, access food and adequate housing, and feel a sense of security. As one participant shared, “When I think about my life in general, a lot of my happiness also stems from my financial independence. I have a great job in a positive and accepting workplace, which allows me to fully account for all my needs, including healthcare and savings. This gives me a sense of security that allows me to breath [*sic*] and appreciate all the other good things in my life” (26, White, cisgender woman, bisexual, pansexual). Not only does this respondent enjoy her job and affirming workplace environment, but she also emphasizes how economic resources, including health insurance and earning enough money to cover needs and savings, provides a sense of stability and positivity. Similarly, another participant connected some benefits of financial access to feelings of happiness, “Having a home, food in my kitchen, and money in my pocket brings me joy” (21, White, gender-expansive person, asexual spectrum, pansexual, queer). Being able to access stable housing, food, and still having money left over supported positive emotion for this participant.

On the other hand, others discussed lacking financial access, which lead to stress and an inability to afford necessities such as housing or medical transition (Table 2). One participant response illustrated the adverse impacts of financial inaccessibility: “When i get a job that gives stable reliable income to get prescribed meds, and a job that actually have [*sic*] equal opportunity to move up the position. i doubt that’s going to happen and am afraid that i’ll be homeless soon again. which is not good for my autism” (29, Asian, transmasculine person, gay). This respondent was looking forward to a future time when they might have a job that provides financial and potentially insurance access, which would allow access to needed medical care, safe housing, stability, career advancement, and peace of mind. Similarly, another exemplar shows a participant who is hoping for the funds to afford gender-affirming care.

Within the context of the US, *Accessing Economic Resources* was a salient facilitating factor that allowed access to everything from enriching pursuits to medical and gender-affirming care to shelter and food. Lack of access here could have an outsized impact on emotional and physical wellbeing.

### Introspective resources

#### Exploring one’s authentic self

*Exploring One’s Authentic Self* referred to the ongoing process of introspection and enaction of one’s genuine identity in spite of the societal, and perhaps familial, expectations placed upon them. While many discussed their authentic self in relation their SGM identities, others also brought in additional salient components of their identity, such as race and ethnicity, cultural and spiritual beliefs, and age. Per one participant, “I can view the world both as the majority see it as that’s how I was raised but can also look at that objectively as I know I am an outsider seeing it. I like having that distance to view the world more objectively and not assume what is going on is always normal or right” (71, Middle Eastern or Northern African, White, cisgender man, gay, same-gender loving). This participant is thoughtfully considering societal norms and determining for themselves what is reasonable and ethical.

This process often included thinking critically about one’s identity, feeling confident and secure in one’s understanding of their own identity, seeing one’s identity in a positive light, and developing compassion towards oneself and others (Table 2): “I am proud to be a gay man of 63… I am better for having been a sexual outlaw. It made me aware of my intolerance, and taught me to be introspective and self-examining… I am more open to change, because I am a gay man who had to live through the 1970’s through 2000’s” (63, White, cisgender man, gay). This participant views his sexual orientation as enriching his life, allowing self-exploration and a sense of flexibility to change.

*Exploring One’s Authentic Self* also included expressing oneself candidly through fashion or aesthetic choices, openness about one’s SGM identities, and subverting cultural norms surrounding gender, relationships, and sexual orientation: “I am most proud of how I can express my gender. I love shopping for clothes that make ME [*sic*] happy, not the clothes that society says I should wear” (19, White, gender expansive person, bisexual). For this participant, the clothing they wear facilitated authentic and cheerful gender expression, despite society’s gendered clothing norms. For another participant, authenticity looked like candidly living as her genuine self: “I can be and act and come across as who I truly am without hiding, a black butch lesbian” (35, Black, cisgender woman, lesbian, queer, same-gender loving).

Many highlighted ways that they are still in the process of becoming their authentic self, whether through introspection, participation in therapy, or accessing gender-affirming care (Table 2): “I’m proud of myself being able to come into my own after being unable to live as myself entirely and being able to reclaim my own personal power, even if it is one bit at a time” (22, White, Hispanic, transfeminine person, straight/heterosexual). This participant has been able to express herself more genuinely and has experienced a process of progressively discovering more about her authentic self.

#### Persevering through Hardship

Many respondents described experiences of adversity, whether ongoing or past. These experiences often related to encountering social rejection and stigma due to their SGM identities. Many discussed ongoing struggles to accept themselves and their SGM identities, as exemplified by the following respondent: “I had trouble coming out due to a religious/conservative upbringing and culture. I remain closeted and am currently unable to work so am going to remain in an outwardly seeming heterosexual relationship. It would be a triumph for me personally to be able to live authentically at long last” (51, Hispanic, cisgender woman, lesbian). This respondent is grappling with religious and cultural values, as well as a current need for financial support. Although this participant has not yet been able to come out, she is persisting with the hope of living authentically in the future.

Other participants reflected on ways they had grown to accept themselves, whether through gender-affirming care, learning more about themselves, or engaging in affirming SGM communities (Table 2): “(If you had asked these questions early in my transition, i would have answered differently: i wanted nothing more than to just ‘be normal’. now i’m many years into my transition and am happy)… I spent 28 years trying to be a Good Daughter, a Good Wife, and live the life i was supposed to live. it was killing me. I finally recognized why i couldn’t make it work, why i couldn’t do it any longer, and finally, for the first time, i’m happy, i’m living honestly, and i’m actually Me [*sic*]” (35, White, transmasculine person, asexual spectrum, queer). Through the process of *Exploring One’s Authentic Self* and transitioning, this participant was able to accept themselves and find contentment.

Adults ages 35 and older, especially those 50+, often discussed their experiences of living through intense anti-SGM stigma and coming out on an extended timeline, as cultural norms shifted: “I spent most of my life in the closet, and came out in my 50’s after a long marriage (hetero) and raising two children. Now I live with my partner (male) and am happier than I have ever been in my life. I am proud of being a gay man” (67, White, cisgender man, gay). For the older respondents, self-acceptance, cultivating affirming intimate relationships, and access to gender-affirming care often occurred later in life.

Additionally, some respondents described ongoing stress related to health concerns, financial concerns, and inaccessibility of gender-affirming care: “Before my post-op surgery, I was miserable inside. I thought of suicide several times a week—at school, in the military, driving down the highway, in the synagogue, at my mom’s house, visiting friends—all the time. all this was due to that driving force within that wanted to express to an outward appearance. Even going through HRT [hormone replacement therapy], the thoughts were still present (thinking that nothing would become of it), even the depression pills rarely worked. Being who I am, I am alive and… I feel much better and can start being myself. Still depressed because I am not what I pictured I would be looking like and not enough funds to complete the total picture (FFS [facial feminization surgery], VFS [voice feminization surgery], BBL [Brazilian butt lift], lipo-suction, and hair implants—life can be trying). I will continue with life and see where it leads?” (65, White, transfeminine person, asexual). This participant has endured extreme hardship and experienced substantial relief through accessing gender-affirming hormones and surgery; however, she remains depressed and unable to access additional gender-affirming procedures that would allow her to ‘complete the total picture.’ Despite these things she is choosing to persist and see what the future holds.

Others described ongoing negative intragroup stigma experiences related to race/ethnicity, transgender status, bisexual identity, body size, and disability. Per one respondent: “I don’t have positive experiences… I’m [a] trans dude (FtM), Asian, Short… (fat), with diagnosed autism [and additional disabilities]… I’m ugly or made to feel ugly [within the SGM community]… I only get weird into disability guy or just not be thought as lgbtqi with disability isn’t a sexual beings [*sic*] within lgbt community. with asian feature or height or whatever i’m doing wrong i can’t attract ppl who really cares” (29, Asian, transmasculine person, gay). This participant describes feeling sexually and romantically undesirable and, at times, fetishized due to a combination of his race, disabilities, and body size. For many, like this participant, the very act of surviving amidst adversity can be conceived of as perseverance. Others described their struggles to navigate predominately white SGM spaces (Table 2). One respondent highlighted that his unique experience as an African American gay man: “I am often assumed to be straight. When people realize that I am a sexual minority, they tend to lump me with White Americans who are sexual minorities. My experiences as an African American gay male are drastically different than my sexual minorities brothers from other racial backgrounds” (53, Black, cisgender man, gay). For this participant his experiences as a gay man occur within and are co-constructed by the US social context of structural racism and anti-Blackness.

## Conclusions

Using constructivist grounded theory, we identified resources that are hypothesized to promote SGM resilience. Specifically, we described eight resources (*Connecting with Others*, *Cultivating Family*, *Helping Others*, *Participating in Culture and Spirituality*, *Engaging in Enriching Pursuits*, *Accessing Economic Resources*, *Exploring One’s Authentic Self and Persevering through Hardship*) that support joy and positive self-conceptions of SGM identity. These resources interact and develop across the life course; as such, resources may look differently for individuals at different life stages.

This model was informed by an intersectional lens and a sampling strategy that amplified voices that have historically been underexplored in SGM research, such as individuals from racial and/or ethnic minority groups, transgender and/or gender diverse individuals, individuals on the asexual spectrum, and older adults. Exploring these diverse perspectives provided a rich basis for our qualitative analysis and allowed the researchers to investigate resources that may promote SGM resilience for individuals of different positionalities. Participant responses revealed experiences like enacted stigma within SGM communities and cohort differences within resources, which may not have been visible within more homogenous samples.

The resources theorized by the model correspond well with previous qualitative coping and resilience research in SGM populations and the population at-large. We identified various important social resources, consistent with many studies that have highlighted the integral role of connecting with community (both SGM and broader) and cultivating family in promoting resilience for SGM youth [18,34], SGM adults [20,21,35], and SGM older adults [24,25]. Additionally, in alignment with our findings, research by Drabble et al. [2018] underscored the supportive role of animal companionship in the interpersonal resources of sexual minority women. Indeed, social support has been theorized as an important component of SGM resilience that helps to attenuate exposure to minority stress [14,36]. Altruism in the form of activism, volunteerism, and mentorship has also arisen in many studies and theories as a contributor to resilience [18,20,32,34,35], consistent with *Helping Others*, which can provide individuals with a sense of purpose and the feeling of making a difference. Activism may also allow SGM individuals to conceptualize their experiences of discrimination and stigma as a result of structural causes [18], thus rebuffing narratives of adversity as a personal failing. The salience of connection to cultural, heritage, or racial background (as in *Participating with Culture and Spirituality*) has been discussed in studies with gay Latino immigrants [21], and in one’s racial/ethnic identity, and through pride in one’s gender identity among transgender people of color [37]. Connection to spirituality, faith, or religion has been discussed as a potential coping resource for SGM individuals that can help support community members through hard times and give them a sense of a higher purpose [35,38,39].

Turning to the affective generative resources, we identified various enriching pursuits that brought pleasure and satisfaction to participants. These fulfilling activities–such as having hobbies and engaging one’s creativity–have been found to support overall well-being for SGM individuals and others [40,41]. Participating in enrichment brings individuals joy, which may help attenuate the effects of stress [12].

Financial access is known to be a positive factor for general wellbeing [41], while restricted access to economic opportunity was identified as an important stressor in Brooks’ original minority stress model [1981]. Within the US, employment or other financial support facilitates access to money and necessities such as health insurance, housing, and food. As such, the impacts of restricted access to economic resources can be considerable, Our findings add to previous research that identified accessing financial resources, and thus accessing health care, as components of resilience for transgender people of color [37,38] and for SGM people more broadly [42]. Similarly, the 2015 US Transgender Survey found that participants who were experiencing poverty and participants with lower educational attainment were more likely to report serious psychological distress, demonstrating that economic access is an important component of wellbeing [43]. Within the current study, even when economic access was not being discussed explicitly, participants mused about many activities or goals that depended on financial access, such as struggling with health-related costs, saving for gender-affirming care, planning for retirement, or planning to travel.

Considering the introspective resources identified in this study, exploring one’s authentic self through self-exploration and a redefinition of what gender and/or relationships entail has been identified as a potential contributor to positive SGM identity and coping with stressors [20,32,44]. Positive SGM identity has been linked to positive emotions and improved social functioning, which are associated with psychological well-being [13,14]. Contrastingly, experiences of stigma related to one’s SGM identity and the health impacts this may have are well documented and explained by the Minority Stress Model [6].

Persevering through minority stress and the iterative process of thriving comes up many times in the literature, especially considering the definition of resilience as endurance through adversity [7,18,25,35]. A notable subset of participants relayed experiences of oppression within and among subgroups of SGM communities. Specifically, some participants described experiences of enacted stigma including racism, cissexism, biphobia, sizeism, and ableism originating from other SGM community members. This highlights the importance of critical, intersectional approaches to SGM health research and promotion that consider structural privilege and oppression in the creation, implementation, and evaluation of all programming. While further exploration of these experiences was not possible due to the use of an existing data set for this analysis and the questions that had been asked of participants, these findings merit further consideration.

Finally, participant responses within multiple resources varied based on the life course. The life course is a framework that considers the ways that historical socio-cultural contexts, timing of social roles (*i*.*e*., youth, partner, parent) and events, interrelationship between people, and human agency interact [45]. Responses highlighted cohort differences in socio-cultural and political contexts, which affected the types and timing of roles that individuals chose to pursue. Specifically, respondents aged 35 and older often described parenting and spousal roles, while also describing a more protracted path to enacting their authentic SGM identities. Additionally, respondents aged 35 and older described living through profound anti-SGM stigma and legislation, which impacted them greatly and may have lengthened the timeline on which they came out. Future research may elaborate the role of the life course and cohort effects in SGM community wellbeing.

### Implications and usability of findings

These findings are intended to highlight resources that may support SGM resilience and are intended to inform public health interventions for the SGM community. Through the amplification of varied community voices, we have created a model that integrates experiences across diverse subpopulations within diverse SGM communities. This work builds on the findings of previous studies exploring coping and resilience within more homogenous SGM communities.

Interventions that seek to bolster SGM community well-being can build upon these findings and emphasize: fostering and maintaining relationships between community members; providing opportunities for activism, volunteerism, and giving back; facilitating access to financial support, healthcare, and gender-affirming care for community members; incorporating different types of enriching activities into programming; and providing spaces and support for SGM community members to work through processes like positive identity development, persevering through adversity, and/or experiences of minority stress.

Indeed, these resources coincide well with existing frameworks of intervention within SGM communities, such as the Transgender Resilience Intervention Model (TRIM) introduced by Matsuno and Israel of the University of California, Santa Barbara [36]. The TRIM identifies group therapies and support groups, mentorship programs and family and/or couples therapy as locations for group level interventions aimed at building relationships between community members, providing opportunities to give back and provide mentorship, and fostering accepting relationships within family relationships [36]. The TRIM also identifies access to gender affirming care, individual therapy and other self-development and acceptance resources as locations for individual level interventions aimed at identity development, self-exploration and actualization [36]. In addition to these group and individual level interventions, the TRIM also emphasizes the need for SGM-specific cultural competency training within healthcare institutions and academic organizations, approaches to decrease in-group discrimination of trans individuals within broader cisgender sexual minority communities, and the importance of social advocacy to address laws, practices and workplace policies that legitimize stigma against trans communities [36].

To provide some examples of salient opportunities, the Trevor Project provides national remote volunteer opportunities performing crisis intervention with SGM youth 25 and under [46]–this not only provides opportunities to give back to the community, but also bolsters SGM positive identity development. Similarly, Black and Pink facilitates a nationwide pen pal program where individuals can correspond with incarcerated SGM people, as well as providing peer-led opportunities to advocate and organize against the prison industrial complex [47]. This organization also fosters community building, activism, and volunteerism as well as supporting SGM community members as they persevere through adversity. Regarding economic access, community organizations such as the San Francisco LGBT Center provide employment services to prepare members of SGM communities for work and facilitate networking with potential employers [48]. In response to the disproportionate impacts that the ongoing coronavirus pandemic has had on systemically marginalized groups in the US–such as SGM people and Black, Indigenous, and Latinx communities–various mutual aid funds have emerged to offer financial microgrants support to these community members [49,50]. Mutual aid funds like these are exceptionally beneficial ways to connect SGM community members with needed financial resources when safe employment is not readily accessible.

In planning any initiative, it will be important to intentionally consider the impacts of structural privilege and oppression on SGM community members. It is integral to include diverse cross-sections of SGM people in the planning process–especially people of minority race/ethnicity, transgender and/or gender-expansive individuals, people with disabilities, and others who experience discrimination and marginalization within the wider SGM community–to ensure that interventions are relevant, accessible, and inclusive. It will also be important to consider the role of the life course, which suggests that different SGM cohorts will have unique perspectives, needs, and interests. For example, interventions directed at SGM community members aged 35 and up may consider including children in the programming or may explore ways to support community members who experienced decades of concealment before they came out.

### Strengths and limitations

This study has many strengths. The stratified, purposive sampling methodology used ensured that the sample was diverse on many axes, including race/ethnicity, gender identity, sexual orientation, age, and region. By amplifying voices that are frequently marginalized, the researchers were able to ensure that various perspectives and lived experiences informed the model, which should lend more external validity to the results. Second, the sample was the largest used for any single qualitative study of resources that promote resilience across SGM communities that the authors reviewed. Our findings aligned well with the results of existing research, which also suggests broad applicability of results.

A limitation to this study is data depth. Rich data, which is detailed and comprehensive (28), is important to develop a thorough analysis within a grounded theory framework. Because many participants’ responses were fairly succinct, this may have limited the ability to undertake an exhaustive analysis of the phenomena explored. However, by including so many participant responses and purposively sampling for longer responses, we were able to generate an informed model of resources that that support SGM resilience. Future research could use focus groups or interviews to explore the salience of these findings with more extensive data. Another limitation to a grounded theory approach using existing survey data is a reduced ability to theoretically sample for emergent codes or to elicit clarifications or further detail from respondents [51]. Future research utilizing primary data is recommended to confirm the model and elaborate on the theoretical boundaries of each resource. A final caveat to acknowledge is that the resources identified are not necessarily exhaustive and represent prominent findings in a diverse data set. As such, this model does not preclude the existence of additional resources that may be particularly salient or visible within more homogenous groups of SGM sub-populations.

### Final summary

This study employed a strengths-based approach to examine three open-ended questions from The PRIDE Study that explored what brings participants joy and how they positively relate to their SGM identity and how uncovered coping resources may contribute to SGM resilience. We presented a model of eight resources that are hypothesized to contribute to SGM resilience. These resources emerged from a large sample of SGM respondents that was purposively enriched for diversity in race/ethnicity, gender identity, sexual orientation, age, and region of residence. This model adds to and affirms strengths-based research focused on coping processes and resilience. This model can be used to inform public health interventions and programming with SGM communities that bolster community resilience.

## Supporting information

S1 Appendix Sampling strategy (Total Sample: n = 315). (DOCX)
