# Supplementary material for: Our pride, our joy: An intersectional constructivist grounded theory analysis of resources that promote resilience in SGM communities
Source: PLoS One. 2023 Feb 3;18(2):e0280787. doi: 10.1371/journal.pone.0280787 (PMC9897522; doi:10.1371/journal.pone.0280787)

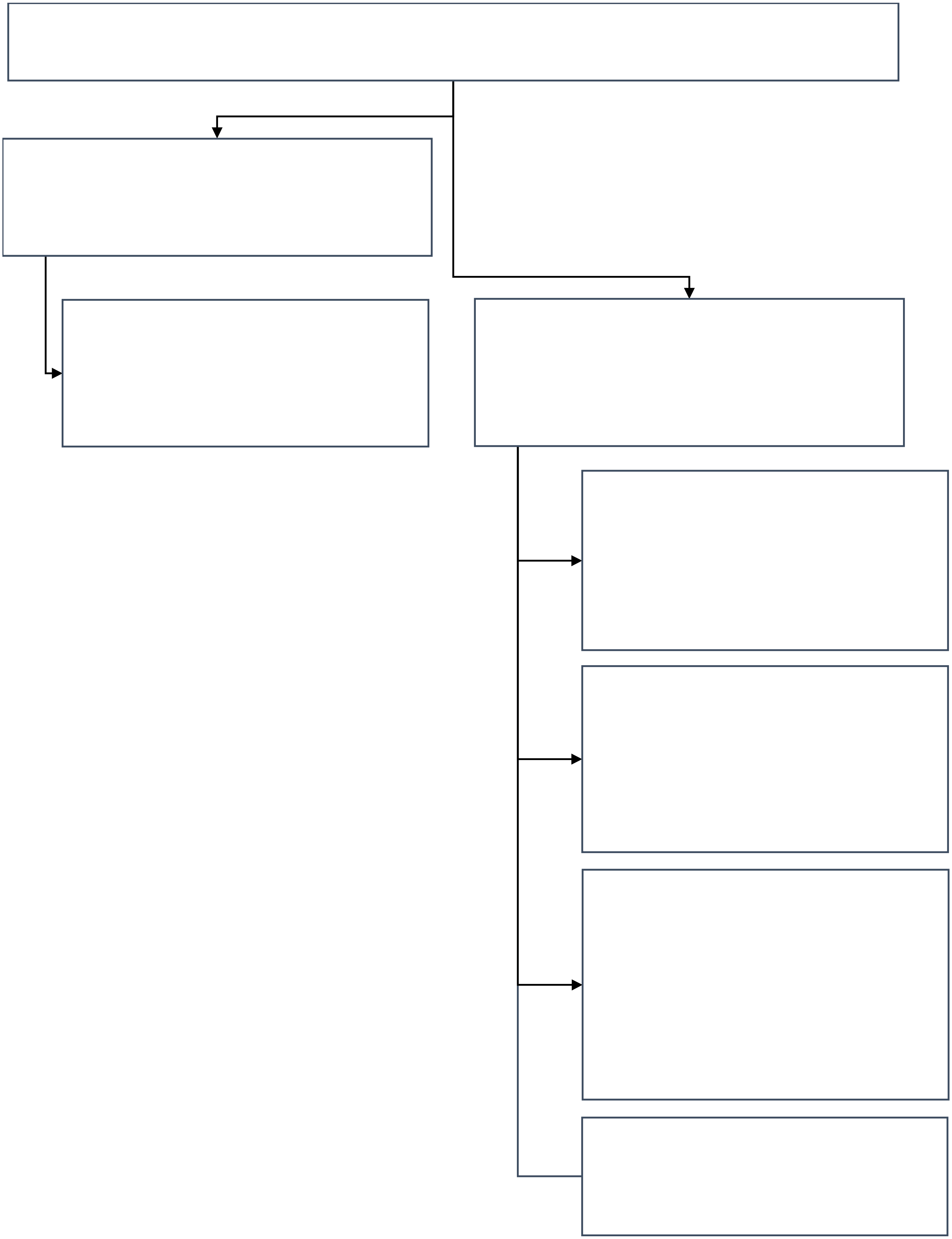

Supplement: Supplementary file 1 [file pone.0280787.s001.docx]
